# Quantitation of next generation sequencing library preparation protocol efficiencies using droplet digital PCR assays - a systematic comparison of DNA library preparation kits for Illumina sequencing

**DOI:** 10.1186/s12864-016-2757-4

**Published:** 2016-06-13

**Authors:** Louise Aigrain, Yong Gu, Michael A. Quail

**Affiliations:** Wellcome Trust Sanger Institute, Wellcome Trust Campus, Hinxton, Cambs, CB10 1SA UK

**Keywords:** DNA library preparation, Next generation sequencing, NGS, Illumina sequencing, Droplet digital PCR

## Abstract

**Background:**

The emergence of next-generation sequencing (NGS) technologies in the past decade has allowed the democratization of DNA sequencing both in terms of price per sequenced bases and ease to produce DNA libraries. When it comes to preparing DNA sequencing libraries for Illumina, the current market leader, a plethora of kits are available and it can be difficult for the users to determine which kit is the most appropriate and efficient for their applications; the main concerns being not only cost but also minimal bias, yield and time efficiency.

**Results:**

We compared 9 commercially available library preparation kits in a systematic manner using the same DNA sample by probing the amount of DNA remaining after each protocol steps using a new droplet digital PCR (ddPCR) assay. This method allows the precise quantification of fragments bearing either adaptors or P5/P7 sequences on both ends just after ligation or PCR enrichment. We also investigated the potential influence of DNA input and DNA fragment size on the final library preparation efficiency. The overall library preparations efficiencies of the libraries show important variations between the different kits with the ones combining several steps into a single one exhibiting some final yields 4 to 7 times higher than the other kits. Detailed ddPCR data also reveal that the adaptor ligation yield itself varies by more than a factor of 10 between kits, certain ligation efficiencies being so low that it could impair the original library complexity and impoverish the sequencing results. When a PCR enrichment step is necessary, lower adaptor-ligated DNA inputs leads to greater amplification yields, hiding the latent disparity between kits.

**Conclusion:**

We describe a ddPCR assay that allows us to probe the efficiency of the most critical step in the library preparation, ligation, and to draw conclusion on which kits is more likely to preserve the sample heterogeneity and reduce the need of amplification.

**Electronic supplementary material:**

The online version of this article (doi:10.1186/s12864-016-2757-4) contains supplementary material, which is available to authorized users.

## Background

Laboratories preparing DNA for Illumina sequencing have access to a quantity of protocols and commercial kits and their numbers are constantly increasing. These kits vary not only in price but also in their protocol. Some of them follow the classical protocol of shearing, end-repair, A-tailing, adaptor ligation and amplification with clean-up between most or all steps, while others have bespoke adaptor ligation steps, or combine several of these steps into a single one, or don’t even require any amplification at all [1, 2]. The nature of the protocol and reagents used might greatly affect the efficiency of the library preparation but very few laboratories conduct a quantitative comparison between several available kits before choosing the most appropriate one for their specific application [3–5].

We developed an assay based on droplet digital PCR (ddPCR) technology to measure the amount of DNA remaining after each steps of a protocol, as well as the percentage of fragment bearing adaptors at their ends after the ligation step, or P5/P7 primers after amplification [6]. In contrast with qPCR, ddPCR doesn’t require the use of any standards to calculate the absolute number of specific molecules in a sample [4, 7–10]. This allows the quantification of not only the overall yield, as normally done with qPCR, but also of the yield of some critical intermediate steps such as the adaptor ligation [11–14].

We present here the quantitative comparison of 9 kits: NEBNext and NEBNext Ultra from New England Biolabs, SureSelectXT from Agilent, Truseq Nano and Truseq DNA PCR-free from Illumina, Accel-NGS 1S and Accel-NGS 2S from Swift Biosciences, and KAPA Hyper and KAPA HyperPlus from KAPA Biosystems. All libraries were prepared using the same DNA sample (barcoded amplicons from phiX174 [15]), and the different kits where compared in terms of overall and stepwise efficiencies, DNA loss, protocol length, flexibility and complexity. We also noticed variations in the size of the final libraries despite the use of identical bead ratio during the clean-up steps. Our results should help laboratories already present or entering the NGS field to choose the most appropriate kit for their specific applications and requirements.

## Results

### DNA library preparation kits for Illumina sequencing

We tested 9 kits listed in Table 1 following the protocol recommended in each manual but keeping the ratio of beads during the clean-up steps, the PCR reagents and settings for the amplification step identical between kits in order to allow a direct comparison between the ddPCR results. We made sure that these slight modifications always remained in the ranges recommended by the manufacturers. Table 2 summaries the overall protocol for each of the kits and the total number of steps required. The total number of steps correlates well with the length of the library preparation both in term of overall preparation time and hands-on time. Combining several steps into a unique one as it is done in the NEBNext Ultra and both KAPA kits not only decrease the overall preparation time, it also improves the DNA recovery as most DNA loss occurs during bead clean-up steps [16, 17]. The KAPA HyperPlus kit also contains a fragmentase step instead of the classical mechanical shearing step and post-shearing clean-up necessary before any other kit [1, 3, 4, 18, 19]. After fragmentase treatment, the sample can go straight into the end repair and A-tailing step, improving the DNA recovery and reducing overall preparation time even further.

**Table 1:** List of the library preparation kits, DNA inputs and adapters tested

Kit	Manufacturer	Reference	DNA inputs (ng)	Adaptors
NEBNext®	New England Biolabs®_Inc._	Cat. #E6040S/L	500	Sanger ([1], current protocols)
NEBNext® Ultra™	New England Biolabs®_Inc._	Cat. #E7370S/L	500	Sanger
SureSelectXT	Agilent	Cat. #930075	500	Sanger
Truseq® Nano	Illumina®	Cat. # FC-121-9010DOC, Part # 15041110 Rev. B	500 & 100	Sanger & Illumina
Truseq® DNA PCR-free	Illumina®	Cat. # FC-121-9006DOC, Part # 15036187 Rev. B	500	Sanger & Illumina
Accel-NGS™ 1S	Swift Biosciences™	Cat. No. DL-ILM1S-12/48, Version 04291444	500 & 100	Swift Biosciences
Accel-NGS™ 2S	Swift Biosciences™	Cat. No. DL-ILM2-48, Version 01131444/2.8	500 & 20	Swift Biosciences
KAPA Hyper	KAPA Biosystems	Cat. #KR0961 – v1.14	500	Sanger
KAPA HyperPlus	KAPA Biosystems	Cat. #KR1145 – v14.1	500 & 20	Sanger

**Table 2:** Description of the type and number of steps for each DNA library kit tested

	End repair	Bead cleaning	A-tailing	Bead cleaning	Adaptor ligation	Bead cleaning	PCR & bead cleaning	Number of steps after shearing
NEBNext	x	x	x	x	x	x	x	8
NEBNext Ultra	2 in 1 ^a^				x	x	x	5
SureSelect	x	x	x	x	x	x	x	8
Truseq Nano	x	x	x		x	x	x	7
Truseq DNA PCR-free	x	xx ^b^	x		x	x		6
Accel-NGS 1S ^c^	Adaptase		1st extension	x	2nd extension	x	x	7
Accel-NGS 2S ^c^	4 different steps + 4 bead cleaning						x	10
KAPA Hyper^d^		2 in 1 ^a^			x	x	(x)	3 (or 5)
KAPA HyperPlus^d,e^			x		x	x	(x)	3 (or 5)

^a^Both End-repair and A-tailing enzymes are combined in a single reaction mix

^b^Illumina recommends performing an upper and lower bead clean-up selection after the end repair step

^c^Swift Biosciences Accel protocols follow different chemical steps than the classical end-repair, A-tailing, adaptor ligation and PCR

^d^KAPA Hyper and KAPA HyperPlus protocol don’t always require a PCR amplification step

^e^KAPA HyperPlus protocol starts with non-sheared DNA. The 1st step of the protocol corresponds to the enzymatic shearing of the DNA sample (fragmentase). This fragmentase step leaves blunt-ended DNA fragments which don’t require End-repair and can go straight to A-tailing without any bead clean-up

Certain kits offer more flexibility than others when it comes to the choice of adaptors. Every kit except the KAPA ones provides their own adaptors, however for most of them the users can decide to use their own if necessary. All the adaptors tested in this study exhibit identical sequence in the first dozen double-stranded bases directly involved in the ligation step, ensuring a similar behaviour independently of the adaptor chosen (Additional file 1: Figure S1). The exception to this is the kits from Swift Bioscience where adaptor ligation is split into 2 sequential steps, on one DNA strand and then the other, making it difficult for the user to use ones’ own adaptors.

### Yields and DNA input

**Fig. 1:**
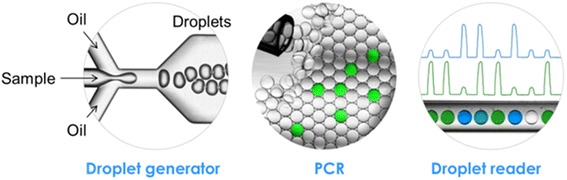
Principle of ddPCR – the droplet generator creates an emulsion with the sample containing the DNA, PCR enzyme and buffer, specific primers and Taqman probe (*left*). Only droplets containing a DNA fragment will exhibit a high fluorescence after the PCR amplification (*middle*). The sample is then analysed with a droplet reader which counts the number of fluorescence and empty droplets in a channel corresponding to the initial number of target molecule in the sample

**Fig. 2:**
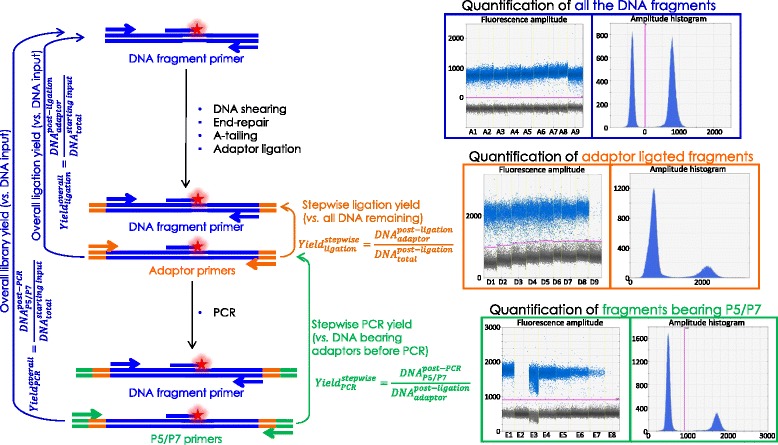
Schematic of the ddPCR assay to test the amount of DNA remaining at each step of the library preparation (using DNA fragment specific primers shown by *blue arrows* [15]) and to measure the amount of DNA fragment bearing adaptors after the ligation step (using the adaptor specific primers shown in *orange*) and P5/P7 primers after the amplification step (using the P5/P7 primers shown in *green*)

**Fig. 3:**
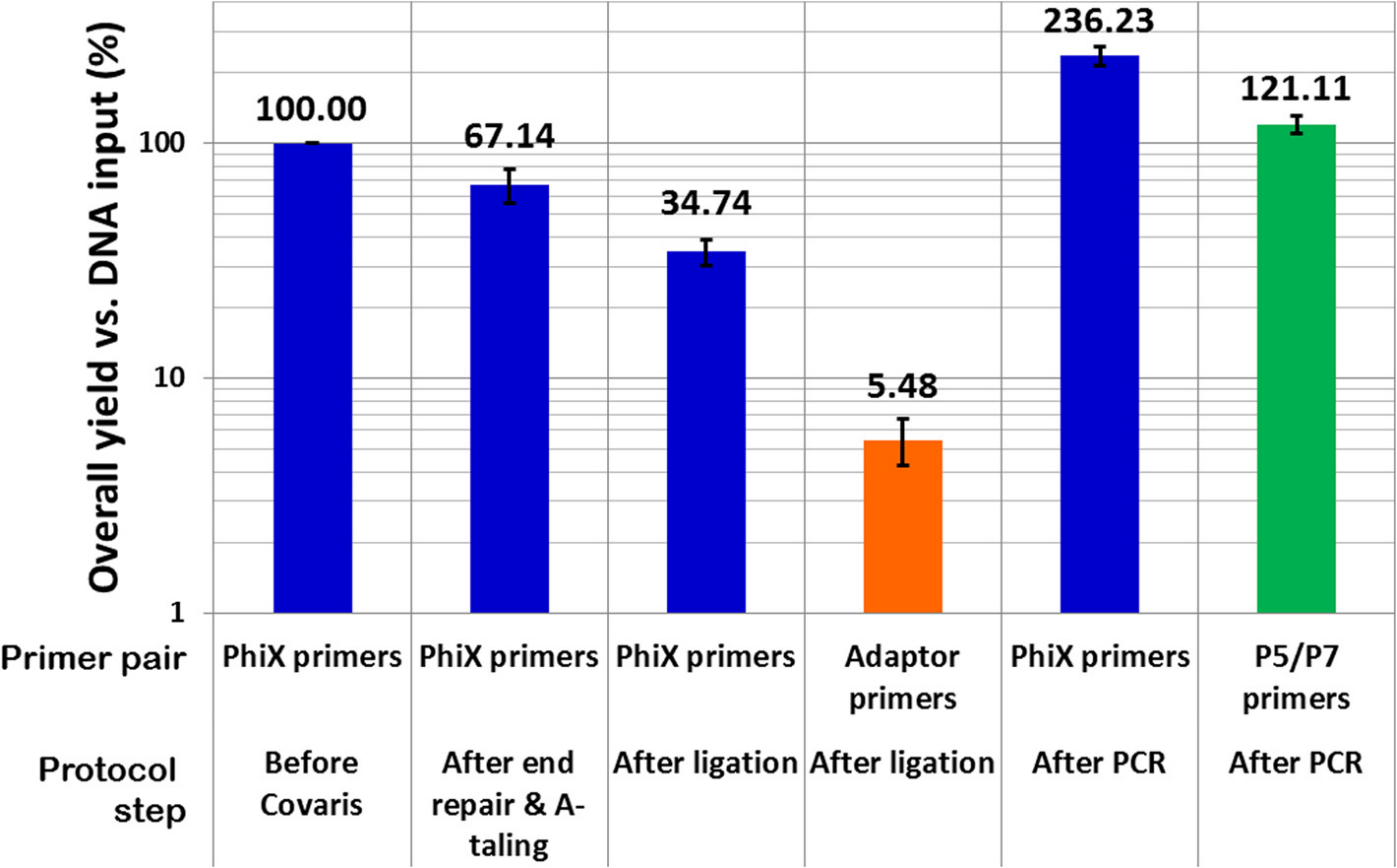
Example of yield measurements obtained with the NEBNext kit when comparing the amount of DNA at each step *versus* the initial DNA input (500 ng). Blue bars correspond to values measured using the PhiX specific primers and reflect the DNA loss at each step mainly due to pipetting and bead clean up. The orange bar corresponds to the value measured with the adaptor specific primers and reflects the amount of DNA in the sample bearing adaptor after the ligation step in comparison with the initial DNA input. The green bar corresponds to the value measured with the P5/P7 primers and reflects the amount of sequencable DNA in the sample at the end of the library preparation

Our droplet digital PCR assay allowed us to probe the amount of DNA remaining in each sample after A-tailing, after adaptor ligation and after PCR (Figs. 1 and 2). We also measured the amount of adaptor ligated DNA after the ligation step and the amount of fragment bearing P5 and P7 primers after the PCR step (Fig. 3). In the case of Truseq DNA PCR-free, the adaptor used already contained the P5 and P7 sequence so that the post-ligation sample is ready for sequencing.

**Fig. 4:**
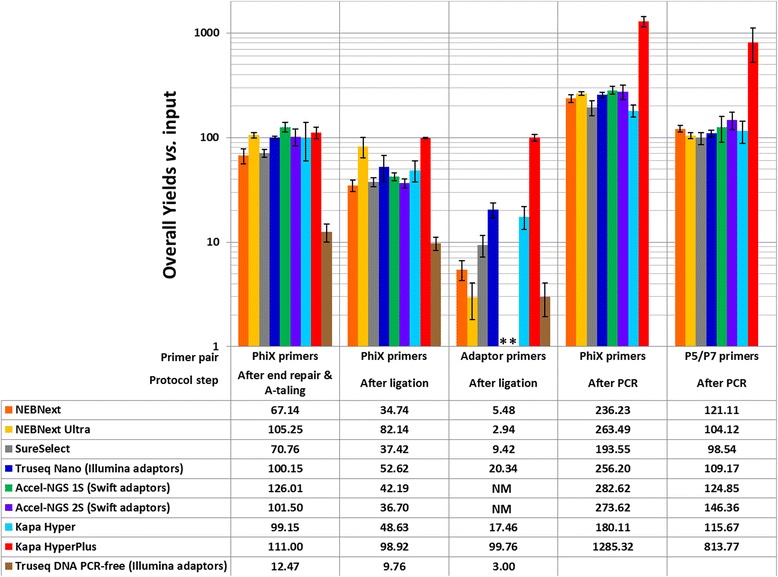
Bar charts showing the overall DNA library preparation yields of the different tested kits in comparison with the initial DNA input (500 ng). Except where mentioned otherwise all libraries were prepared using the original Illumina Paired end adaptor (also named Sanger adaptors) [6, 22]. After end repair and A-tailing, the DNA loss was estimated using the PhiX specific primers (*1st column*). After adaptor ligation, both the DNA amount (PhiX primers, *2nd column*) and the adaptor ligation efficiency (adaptor specific primers, *3rd column*) were measured, except for the Accel kits for which we were not able to measure directly the adaptor ligation efficiency as the adaptor sequences were unknown and are marked by a start (NM for not measured). After PCR, both the total amount of DNA (PhiX primers, *4th column*) and the amount of DNA bearing P5 and P7 primers at their ends were measured (P5 & P7 primers, *last column*)

During all the steps before ligation, low or no DNA loss is observed except with the Truseq DNA PCR-free kit where more than 80 % of the initial DNA was lost due to more numerous and stringent bead clean-up steps recommended (upper and lower Spri clean-ups, Fig. 4) [17]. This explains why the user is advised to start with 1 μg of DNA for the Truseq DNA PCR-free protocol.

After adaptor ligation, we were able to both probe the amount of DNA remaining and the efficiency of the ligation reaction itself, which, as expected, was the most critical step of all. Unfortunately, in the case of the Swift Biosciences kits, we were not able to measurement the amount of DNA bearing adaptors at their ends due to the specificity of the Swift Biosciences adaptor ligation chemistry which prevented us from using our own adaptors and primers. For the other kits, the variation of ligation efficiency was very marked; some kits exhibiting such low adaptor ligation yields that it could impair the final complexity of the library (Fig. 4), while other performed extremely well. This remains the case even when looking at the yield in a stepwise manner (Additional file 1: Figure S2) rather than the overall yield. For NEBNext, SureSelectXT, Illumina Truseq Nano and KAPA Hyper, the ligation step yield varies from 15 to 40 %. A very low step yield of 3.5 % was measured for NEBNext Ultra and in contrast 100 % ligation efficiency was observed for the KAPA HyperPlus kit.

Such variation of ligation efficiencies can be entirely masked when focusing on the post PCR yields. Most kits exhibiting an overall post PCR yield between 100 and 150 % after 10 cycles of amplification when measuring the amount of fragment bearing P5 and P7 primers *versus* DNA input, at the exception of the KAPA HyperPlus kit for which the overall PCR yield is just above 800 %. However the stepwise yields of the PCR steps, when comparing with the amount of DNA bearing adaptors just after the ligation, were much more variable with values ranging from 500 % to almost 4000 %. The yields of the PCR step also appeared anticorrelated with the yield of the ligation step.

For kits designed specifically for low DNA input, we tested the same DNA input as for any other kits, 500 ng, and compared with lower inputs (100 ng or 20 ng). We noticed that the ligation step was slightly more efficient with the higher DNA input, however the same high DNA input led to lower PCR step yields (Additional file 1: Figure S3). High DNA input PCR can indeed inhibit the amplification reaction explaining the anticorrelation observed between ligation and PCR yields; very efficient ligation steps leading to high DNA input for the PCR step. Other factors such as limiting dNTPs or primers during amplification might also have a similar effect.

### Bias on the fragment size

During this study, sample fragment sizes were assayed using a Bioanalyzer to check the profile of the input DNA (same DNA stock for all the samples) and the final libraries [20]. We noticed that the profile of libraries prepared with different kits varied significantly despite the fact that both the DNA input and the bead clean-up ratios were kept identical (except for the Truseq DNA PCR-free kit which recommends an upper and lower Spri clean-up after end repair). All the libraries were started with an equimolar ratio of the 3 PhiX DNA fragments used and we expect some slight variation after the library preparation as the smallest DNA fragment might be prone to more loss during the bead clean-up steps. But the variation observed between kits was much more serious than just loss of the shorter fragments as it can obviously be seen when looking at the example of Bioanalyzer traces in Additional file 1: Figure S4.

**Fig. 5:**
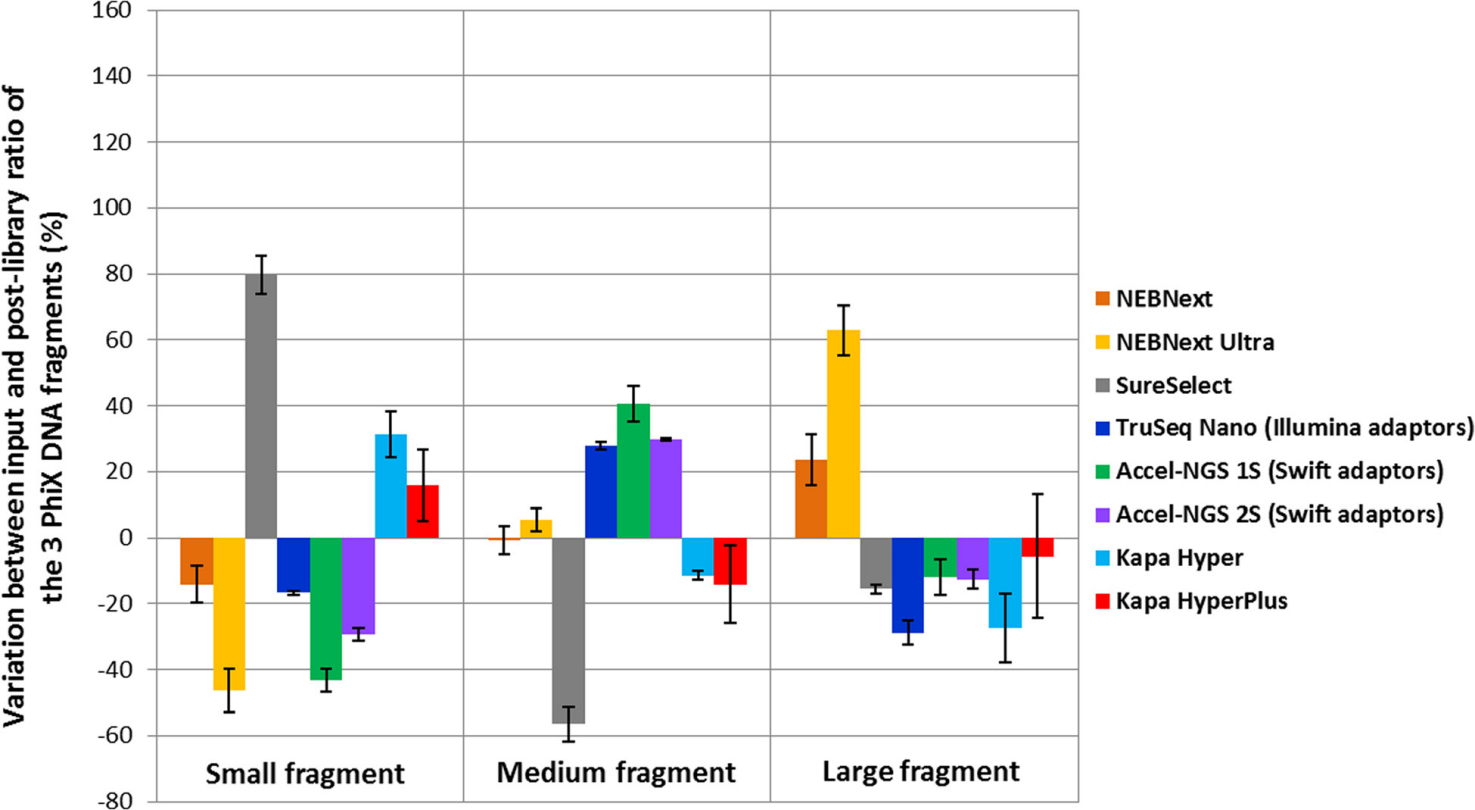
Bar chart representing the percentage of variation between the 3 different PhiX fragments before and after library preparation with the different kits tested. For each sample, the molar concentration of the 3 PhiX fragments was estimated before and after library preparation on a Bioanalyzer chip. The ratio between the 3 fragments before library prep was close to 33 % for each, but important variations were observed after library preparation. Here we plotted the difference between the pre- and post-library preparation ratio as a percentage. All the libraries were prepared using the original Illumina Paired end adaptor [6, 22] except for the Truseq Nano kit and the Swift kits

To quantify this variation more accurately we calculated the ratio between the 3 PhiX fragments before library preparation (equimolar ratio of ~33 % each) and post library preparation. We then plotted the variation between the pre- and post-library preparation ratios as show in Fig. 5. The libraries prepared with the Truseq DNA PCR-free kit were not included in Fig. 5 due to the difference in the protocols which prevent us from doing any straight comparison.

### Data quality

**Fig. 6:**
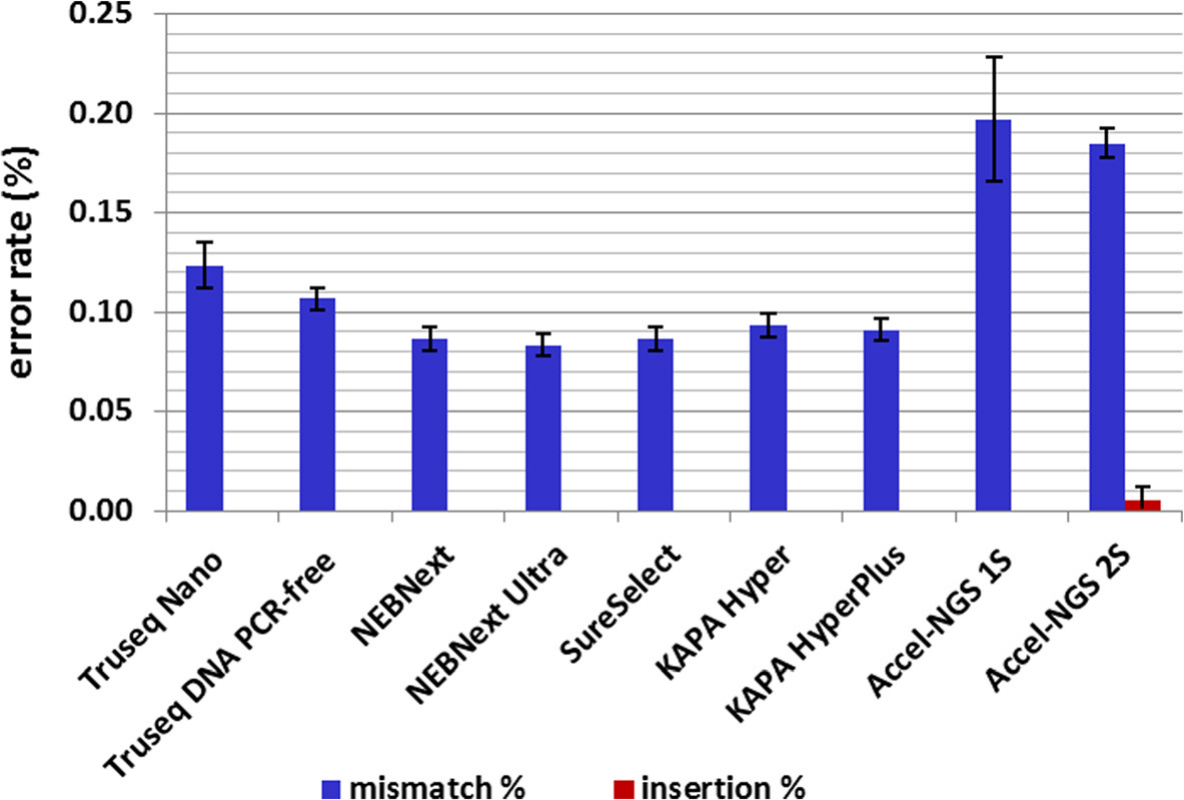
Comparison of sequencing data quality between libraries prepared with different DNA library preparation kits. Deletions were not detected while insertions (*red bars*) were extremely low for all kits. The most common source of error is mismatches (*blue bars*) which vary between 0.08 % and 0.2 % depending on the kit used

All the libraries prepared during this study were sequenced on an Illumina Miseq platform. To compare the data quality of different libraries, we compared the error rates such as insertions and mismatches (Fig. 6). While all libraries performed well with overall error rates lower than 0.2 %, we observed some differences between kits. Both Accel kits exhibit higher error rates than the other, above 0.18 % while all the other kits lead to error rates below 0.13 %. The main source of error for all the kits was always mismatches however, in the case of Accel-NGS 2S kits, insertions were also observed. Among the other kits, the NEB, Agilent and KAPA ones had the best performance with error rates below 0.1 %.

## Discussion

We compared the practicability, reproducibility and quality of the libraries and sequencing data produced using 9 different kits to prepare Illumina DNA libraries. What we mean by practicality is the overall time required to prepare a library, the hands on time, and the number of steps involved in the process [1, 3, 4]. In our experience, overall preparation time correlates very well with the total number of steps in a protocol when including clean-up steps. Therefore any kit combining several steps into a single ones and limiting the number of clean-ups should be favoured if preparation time is a critical parameter in the project. The fastest protocols are the NEBNext Ultra kit and the KAPA kits, particularly the KAPA HyperPlus, and up to certain extend the Illumina Truseq DNA PCR-free.

Certain kits are specifically designed for low DNA input such as the NEBNext Ultra and Swift Accel-NGS 2S, while others such as the KAPA ones accept a wide range of DNA input from a 1 ng to 1 μg. However if the ligation efficiency of a kit is very low (<15 %) as it is the case for the NEBNext Ultra kit, or if the DNA loss during the library preparation is high (>50 %) as it is the case for the Accel kits, the final amount of sequencable DNA becomes worryingly low. It is important to highlight that this study focuses on evaluating the efficiency of each steps of different library preparation protocols and we did not assess directly the complexity of the library. Bearing this in mind, the KAPA HyperPlus kit which exhibits a fully efficient adaptor ligation step and less than 10 % DNA loss, appears as the kit of choice for any low DNA input sample.

The Truseq DNA PCR-free kit is the only one recommending an input as high as 1 μg due to the stringent clean-up steps to remove both too long and too short DNA fragments from the library. Nonetheless avoiding any amplification step presents great advantages not only in terms of preparation time but also to minimise bias. The amplification step can indeed introduce artificial mutations which are difficult to distinguish from real SNPs [1, 21, 22]. The sample composition can also be affected by polymerases amplifying preferentially certain fragments over others, and this phenomenon can become very preeminent for non-GC neutral samples [13, 23–25]. Although certain enzymes have been shown to exhibit very high fidelity and low bias even for AT- or GC-rich DNA, the possibility to simply avoid any amplification at all can drastically improve the data quality for such samples [1, 2]. It is important to highlight that not only the Truseq DNA PCR-free kit but also any other kit exhibiting a high ligation efficiency could potentially be used without any PCR step, as long as the sequence of the used adaptors contains the P5/P7 primers sequence necessary for sequencing on an Illumina platform.

Another factor often ignored is the shearing step. Most protocols necessitate already sheared and cleaned-up DNA to start the library preparation, and sonication on a Covaris instrument is often the method of choice due to its reproducibility and tunability [1, 3]. Enzymatic shearing presents several advantages such as low cost (no need to invest in neither a specific instrument nor consumables) and low DNA loss (samples can go straight from enzymatic shearing to end-repair without any intermediate clean-up step) [4]. However until recently most enzymatic shearing mix available exhibited high bias toward certain GC content samples and difficulties to control the average DNA fragment size in a library. But the latest generation of enzymatic shearing mixes such as the fragmentase provided with the KAPA HyperPlus kit appears much more reliable, controllable and less susceptible to bias (Additional file 1: Figure S5, [26]). KAPA HyperPlus isn’t the only kit using such a streamlined protocol and subsequently we have tested other kits such as the NEB UltraII that also exhibits very high ligation yields in early testing (>85 %, data not shown).

We observed an interesting phenomenon when comparing the ligation and PCR yields of the different kits as both appear almost anticorrelated in our data (Fig. 4 and Additional file 1: Figures S2 and S3). An explanation could be that when the initial DNA input is low or when the ligation step efficiency is poor, the amount of adaptor ligated DNA going into the PCR reaction is very little; on the other hand if the adaptor ligation is very efficient or the starting DNA input very high, important amount of DNA is going into the PCR reaction. Yet high DNA substrate isn’t recommended for PCR reactions as it is known to inhibit the amplification reaction. Such phenomenon can hide differences between kits since a protocol exhibiting high ligation efficiency will produce a high concentration of PCR substrate (adaptor ligated fragments) which can inhibit amplification, while on the other hand a kit exhibiting low ligation efficiency will lead to a very efficient PCR (no substrate excess), both kits giving similar amount of final library product. A high ligation yield insures the preservation of the sample diversity and decreases the amount of amplification required, avoiding the introduction of additional bias during PCR [27]. In that respect the Illumina Truseq Nano and PCR free kits, as well as the KAPA Hyper kit exhibited some of the highest ligation yields, above 30 %, and the ligation step with the KAPA HyperPlus was fully efficient.

Finally we noticed variations in the ratios of our 3 control amplicons in the final libraries when prepared with different kits. We cannot discriminate the two possible sources of variation, fragment size or fragment sequence, and both are most probably playing a role here. To avoid introducing any bias in our comparison, we used the same Spri ratio during the clean-up steps with every kit tested except Truseq DNA PCR-free. However the same Truseq Nano kit resulted in very different fragment ratios when using the Sanger adaptors [1] rather than the Illumina adaptors (royal and navy blue bars in Fig. 5) implying that the sequence of the adaptors and of the DNA fragments involved in the library preparation does play a role and might introduce certain bias. The kits leading to the lowest variations (<25 % for each fragment size) and therefore probably introducing the least bias were KAPA HyperPlus and NEBNext.

## Conclusion

We identified the kits that are the most practical and the most efficient, both characteristics often working hand in hand. Using a novel ddPCR assay, we were able to deconvolute the influence of each intermediate step in the library preparation and highlight the significance of adaptor ligation efficiency which can be hidden when focusing only on the overall library preparation yield after amplification. Unlike qPCR measurements [11, 12], our ddPCR assay doesn’t require any specific standards and can be used to assess the efficiency of any other kit or protocol not mentioned in this study or not realised yet, providing a great tool for direct comparison and objective selection. The emergence of PCR free protocols and simplified protocols merging several steps into one will certainly improve not only the workflow, overall and hand on times of DNA library preparation, but also the chemical efficiency of these.

## Method

### DNA sample

All the libraries compared in this study were prepared with the same DNA sample stock. The sample consisted of three amplicons of different sizes but sharing some homologous sequence from PhiX174 (214 bp, 397 bp and 568 bp, see Table 3) [15].

**Table 3:** Description of the primers and Taqman probe used in ddPCR assay

Oligonucleotide	Sequence	Comments
PhiXa sens	GGC GCT CGT CTT TGG TAT GTA	Amplification and detection of 214 bp fragment
PhiXb sens	TGA ATT GTT CGC GTT TAC CTT	Amplification of 397 bp fragment
PhiXc sens	GTA CGC TGG ACT TTG TAG GAT	Amplification of 568 bp fragment
PhiX rev	GGC GTC CAT CTC GAA G	Amplification and detection of all 3 DNA fragments
Adaptor sens	CTT TCC CTA CAC GAC GCT CTT	Detection of adaptor ligated fragments
Adaptor rev	ATT CCT GCT GAA CCG CTC TTC	Detection of adaptor ligated fragments
P5 primer	AAT GAT ACG GCG ACC ACC GA	Detection of final library fragments
P7 primer	CAA GCA GAA GAC GGC ATA CGA	Detection of final library fragments
Taqman probe	[6FAM]GCGATAACCGGAGTAGTTGAAATG[TAM]	Taqman probe targeting the common sequence between the 3 DNA fragments

### DNA library preparation kits for Illumina sequencing

In this study, the following kits were tested and compared: NEBNext and NEBNext Ultra from NEB, SureSelectXT from Agilent, Truseq Nano and Truseq DNA PCR-free from Illumina, Accel-NGS 1S and 2S from Swift Biosciences, and KAPA Hyper and KAPA HyperPlus from KAPA Biosystems (see Table 1). All kits where tested with 500 ng DNA input and the ones designed specifically for low input DNA were also tested with lower amount of staring material (see Table 1). All samples were processed in triplicate and the error estimations of our values correspond to the standard deviations calculated on each triplicate set. We followed closely the manufacturers recommended protocol for each kit as well as the amount of adaptor added to the sample prior to ligation in correspondence to the DNA input used. For the sake of consistency and to allow an objective comparison between libraries, all the libraries which underwent a PCR step, independently from the kit used, where amplified using the KAPA HiFi Master Mix (KR0370 – v5.13) and P5/P7 primers following precisely the protocol and program recommended by KAPA for 6 amplification cycles.

In order to mimic the preparation of a normal genomic DNA library, the DNA stock was sheared using a Covaris S200 (settings for 500 bp peak as recommended by the manufacturer) and clean-up with a 1.8:1 beads:DNA ratio before starting the library protocol following the kit manuals. The only exception was for the KAPA HyperPlus kit which contains is own enzymatic shearing step. In this specific case, we followed the recommended protocol without any initial Covaris shearing and incubating the DNA with the fragmentase mix for 5 min at 37 °C.

### Droplet digital PCR (ddPCR) assay

In order to evaluate the efficiency of each library preparation, we developed an assay based on droplet digital PCR technology [4, 7, 28–30]. All the measurements are done on a Bio-Rad QX200 instrument. Samples are diluted and mix with recommended ddPCR master mix, and with specific primers and Taqman probe targeting the homologous region of our amplicons (Table 3). An example of the precise dilutions required for a library starting with 500 ng DNA input is given in the Additional file 2: Table S1 and typically varies between 10^5^ and 10^7^ depending on the library preparation step and the specific reaction volume at this step. The dilutions were decreased accordingly for lower input libraries (5 times less for 100 ng input, 25 times less for 20 ng input). We always aimed for maximal number of molecules per ddPCR reaction of 10,000. The ddPCR aqueous reaction mix is then converted on the Droplet Generator into an emulsion containing tens of thousands of droplets containing either zero or a single DNA fragment due to the very low dilution.

The ddPCR program correspond to the following setting with a temperature ramping of 2 °C/s: denaturation for 10 min at 95 °C, then 40 cycles for denaturing for 30 s at 94 °C and annealing/extension at 65 °C for 60 s, and a final enzyme deactivation at 98 °C for 10 min. After PCR, only droplets initially loaded will exhibit high fluorescence due to the annealed Taqman probe allowing the counting of the number of molecules in the initial sample by the droplet reader without the necessity of any standards (Fig. 1) [31]. Each measurement was done in triplicate.

To evaluate the amount of DNA remaining after each step as well as the yield of the reactions, two independent measurements are carried out: the amount of overall molecules remaining in the sample at each steps in the protocol (after A-tailing, after adaptor ligation and after PCR) using primers targeting the homologous sequence of the DNA fragments and the amount of molecules bearing adaptors after ligation or P5/P7 primers after PCR amplification, this time using adaptor specific and P5/P7 primers (Table 3*,* Fig. 2). One advantage of the ddPCR method is that it doesn’t depend on equivalent PCR efficiencies for each measurement as it gives a binary answer for each droplet [31]. The critical point is to insure a clear distinction between loaded and empty droplets fluorescence intensities (Fig. 2).

The DNA loss and chemical yield of each steps and of the overall library preparation are calculated by combining the different ddPCR measurements of the total DNA remaining at a certain step, the adaptor ligated DNA or the final library bearing P5/P7 adaptors at their ends. For the first steps of the library preparation, DNA shearing, end repair and A-tailing, only the DNA loss due to bead clean-up is measured. However both DNA loss and chemical efficiencies are calculated for the last 2 steps of each protocols, adaptor ligation and DNA amplification. It is important to highlight that the ligation yield corresponds here to the overall yield of all the previous chemical steps up to the ligation, including end repair and A-tailing, so variations in ligation yield between protocols might also reflect difference in the end repair or the A-tailing steps rather than just the ligation itself.

### Yield calculations

In this study, we distinguish the “overall yield” of a library preparation protocol step from the “stepwise yield”. The overall yield corresponds to the amount of DNA remaining after a certain step in comparison with the initial DNA input of the library preparation (500 ng, 100 ng or 20 ng depending on our samples). The stepwise yield corresponds to a measurement of the efficiency of a chemical step itself by comparing the number of molecules being successfully transformed (for example the number of molecules bearing adaptors on both ends after the adaptor ligation step) with the total number of molecules remaining in the sample (in our example the total number of molecule after ligation regardless of the presence of adaptor is measured by ddPCR using the PhiX primers). The comparison of the overall yield and stepwise yield for an identical step allows us to deconvolute the amount of DNA loss, simply due to bead clean-ups and pipetting, from the actual efficiency of a chemical step such as the ligation of adaptors. More details on the overall yield and step yield calculations can be found below and in the Additional file 2: Table S1 and below.

Overall yields are calculated as a ratio of the number of DNA molecules left at a certain step of the library preparation protocol (for example *DNA*_*adaptor*_^*post* − *ligation*^ for the DNA amount left after ligation and bearing adaptors measured with adaptor primers) *versus* the initial DNA input (*DNA*_*total*_^*starting input*^ measured with PhiX primers, Figs. 2 and 3 and Additional file 2: Table S1):

$$ Yiel{d}_{ligation}^{overall}=\raisebox{1ex}{$DN{A}_{adaptor}^{post- ligation}$}\!\left/ \!\raisebox{-1ex}{$DN{A}_{total}^{starting\  input}$}\right. $$

The efficiency of a specific step, stepwise yield, for a sample prepared with a specific protocol is calculated by comparing the overall number of DNA molecules remaining in the sample just after this step (for example for the ligation stepwise Yield *DNA*_*total*_^*post* − *ligation*^ measured with the PhiX specific primers, Fig. 2) with the amount of DNA fragments bearing adaptors at their ends in the very same sample (for our example of ligation step yield, *DNA*_*adaptor*_^*post* − *ligation*^ measured this time with the adaptor specific primers, Fig. 2 and Additional file 1: Figure S2 and Additional file 2: Table S1):

$$ Yiel{d}_{ligation}^{stepwise}=\raisebox{1ex}{$DN{A}_{adaptor}^{post- ligation}$}\!\left/ \!\raisebox{-1ex}{$DN{A}_{total}^{post- ligation}$}\right. $$

### Sequencing and data processing

Libraries were multiplexed in batches of 15 and sequenced on an Illumina Miseq instrument with the V2 chemistry. Runs were 150 base paired-end reads and the appropriate single index read.

After sequencing, reads were mapped with the reference using BWA [32]. Then base errors were counted throughout the mapped reads for mismatches, insertions and deletions and the error rates were obtained by averaging them with the total number of bases in the mapped region of all reads.

## Abbreviations

bp, base pairs; ddPCR: droplet digital PCR; NGS, next-generation sequencing; PCR, polymerase chain reaction; Tm, melting temperature

## Additional files

Additional file 1: Figure S1. Comparison of the overall yields for libraries prepared with the Truseq Nano kit with either the Sanger adaptors (original Illumina adaptors, in pink) and the modern Illumina adaptors (in blue). **Figure S2** Bar charts showing the stepwise DNA library preparation yields of the different kits tested. Initial DNA input: 500 ng. Except where mentioned otherwise all libraries were prepared using the original Illumina Paired end adaptor (Sanger adaptors) [6, 22]. The most critical steps correspond to the adaptor ligation for which the yield varies from 3.50 to 100 % depending on the kit tested. **Figure S3** Bar charts showing the comparison of the overall DNA library preparation yields of the different kits tested depending on the initial DNA input. Although higher DNA inputs lead to slightly higher adaptor ligation yields, the final PCR yield appears much greater when the initial DNA input is low. **Figure S4** Bioanalyzer traces of 3 libraries prepared with the PhiX amplicons of 3 different sizes. The initial input sample contained a equimolar ratio of the 3 amplicons whereas this ratio varies in the final libraries presented here depending on the kit used (Truseq Nano in red, SureSelect in blue and KAPA hyper in green). **Figure S5** Enzymatic shearing using the fragmentase provided with the KAPA HyperPlus kit. A) Tunability and robustness of the fragmentase treatments depending on the GC content of the DNA sample, DNA input and the incubation time. B) KAPA HyperPlus libraries GC contents and their correlation with the theoretical values. (PPTX 367 kb) Additional file 2: Table S1. Example of dilution factors and yield calculations for the NEBNext libraries with 500 ng DNA input and Sanger adaptors. The calculation of the number of molecules measured by ddPCR at each step of the library preparation is described in the first 7 columns. The number 20 in column 7 corresponds to the ddPCR reaction volume in μL as only 1 μL of diluted sample is pipetted in the final ddPCR reaction mix of 20 μL total. Column 8 to 10 describe the calculation of the Overall Yield of each steps whereas column 11 to 14 (table split on 2 pages) explain the calculations of the Step Yield. The equations corresponding to each cell/column values are displayed in blue. (DOCX 22 kb)
