# Supplementary material for: Quantitation of next generation sequencing library preparation protocol efficiencies using droplet digital PCR assays - a systematic comparison of DNA library preparation kits for Illumina sequencing
Source: BMC Genomics. 2016 Jun 13;17:458. doi: 10.1186/s12864-016-2757-4 (PMC4906846; doi:10.1186/s12864-016-2757-4)
Supplement: Additional file 1: Figure S1. — Comparison of the overall yields for libraries prepared with the Truseq Nano kit with either the Sanger adaptors (original Illumina adaptors, in pink) and the modern Illumina adaptors (in blue). Figure S2 Bar charts showing the stepwise DNA library preparation yields of the different kits tested. Initial DNA input: 500 ng. Except where mentioned otherwise all libraries were prepared using the original Illumina Paired end adaptor (Sanger adaptors) [6, 22]. The most critical steps correspond to the adaptor ligation for which the yield varies from 3.50 to 100 % depending on the kit tested. Figure S3 Bar charts showing the comparison of the overall DNA library preparation yields of the different kits tested depending on the initial DNA input. Although higher DNA inputs lead to slightly higher adaptor ligation yields, the final PCR yield appears much greater when the initial DNA input is low. Figure S4 Bioanalyzer traces of 3 libraries prepared with the PhiX amplicons of 3 different sizes. The initial input sample contained a equimolar ratio of the 3 amplicons whereas this ratio varies in the final libraries presented here depending on the kit used (Truseq Nano in red, SureSelect in blue and KAPA hyper in green). Figure S5 Enzymatic shearing using the fragmentase provided with the KAPA HyperPlus kit. A) Tunability and robustness of the fragmentase treatments depending on the GC content of the DNA sample, DNA input and the incubation time. B) KAPA HyperPlus libraries GC contents and their correlation with the theoretical values. (PPTX 367 kb) [file 12864_2016_2757_MOESM1_ESM.pptx]

## Slide 1
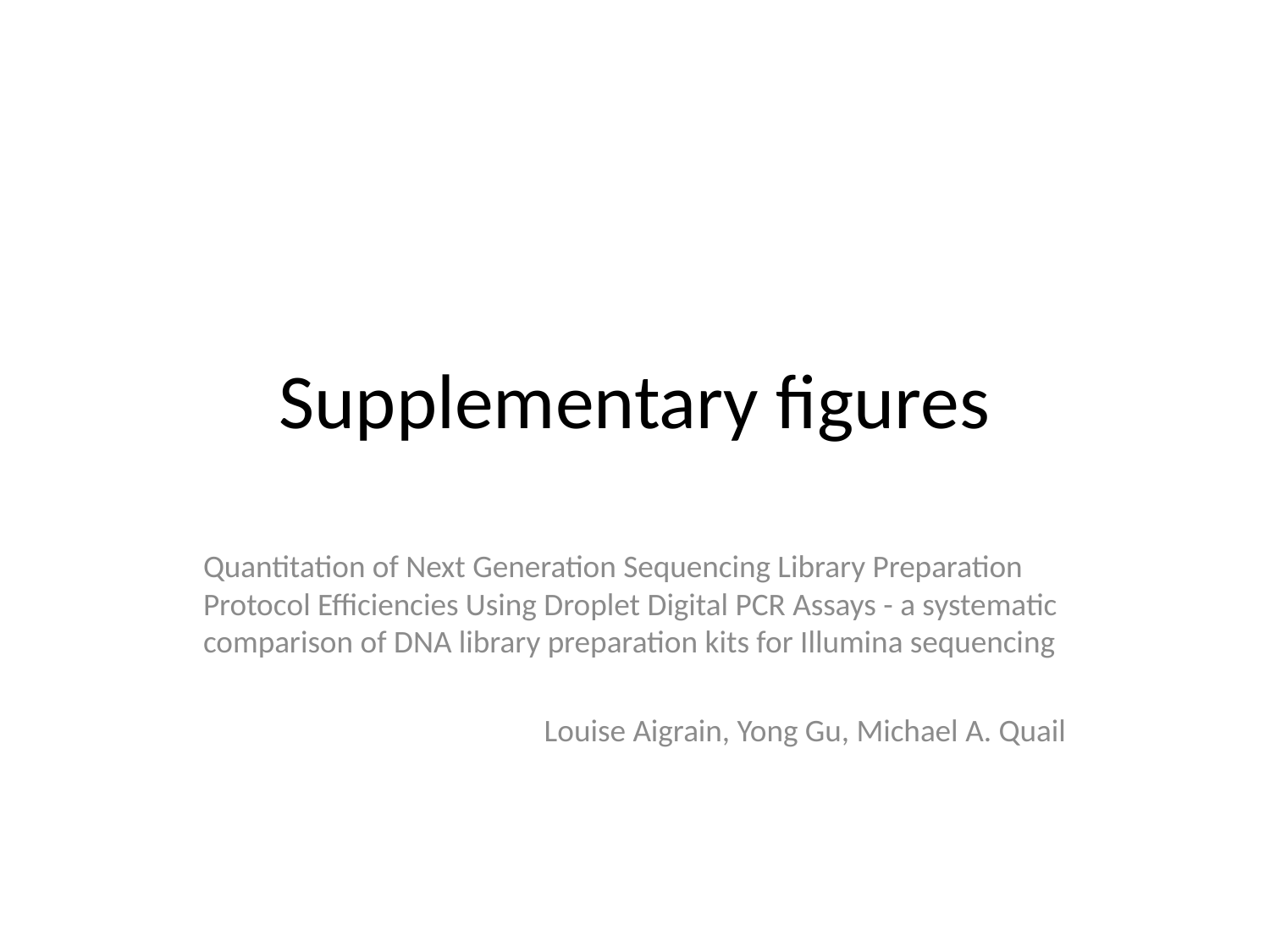

# Supplementary figures
Quantitation of Next Generation Sequencing Library Preparation Protocol Efficiencies Using Droplet Digital PCR Assays - a systematic comparison of DNA library preparation kits for Illumina sequencing
Louise Aigrain, Yong Gu, Michael A. Quail

## Slide 2
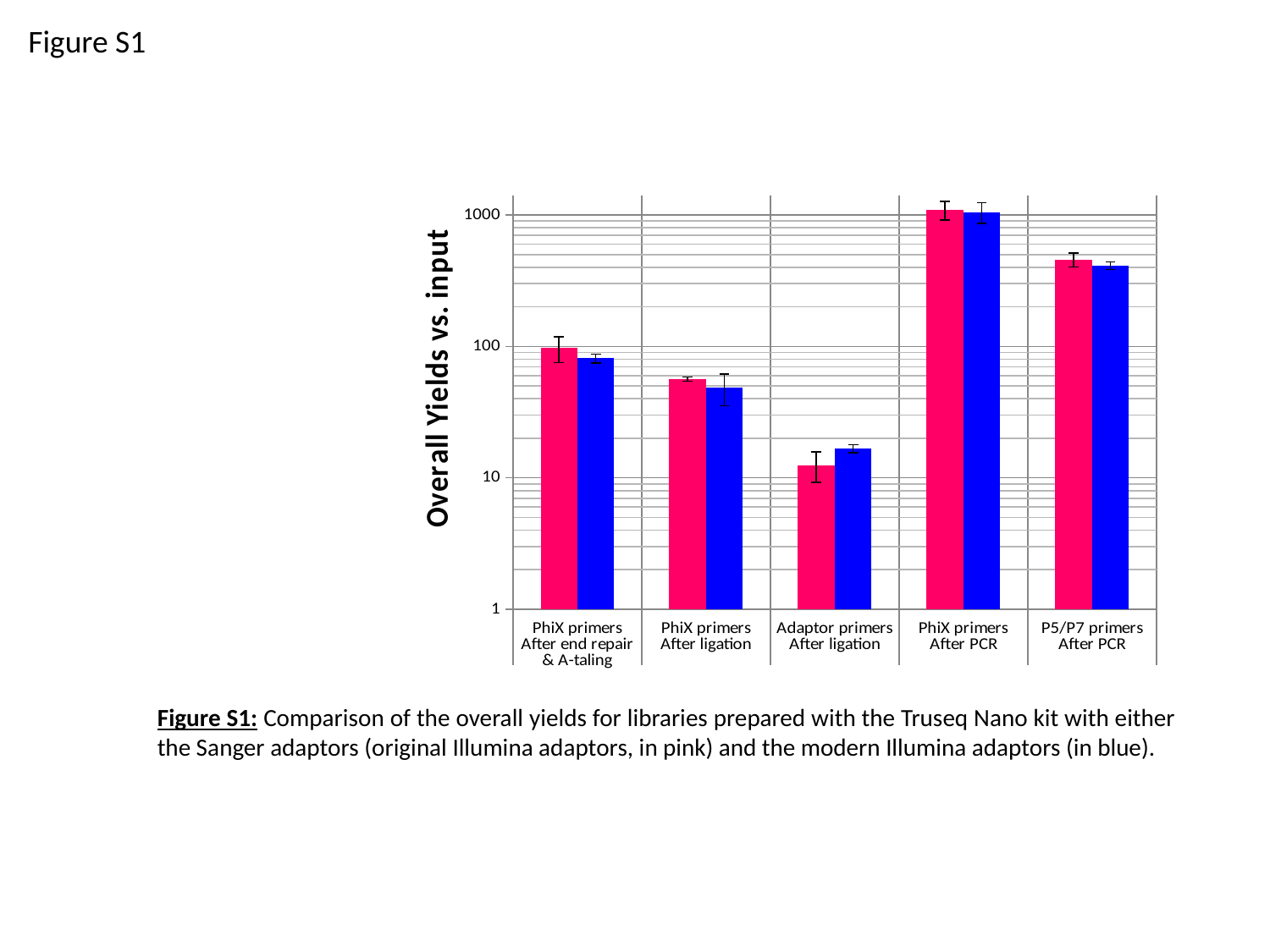

Figure S1
### Chart:
| Category | Truseq Nano (100 ng input, Sanger adaptors) | Truseq Nano (100 ng input, Illumina adaptors) |
|---|---|---|
| PhiX primers | 96.84469898677692 | 81.24160243484647 |
| PhiX primers | 56.50648664233339 | 48.56751797213739 |
| Adaptor primers | 12.493406460701449 | 16.72105300488217 |
| PhiX primers | 1091.4867745681306 | 1050.3244877051188 |
| P5/P7 primers | 456.75809788704834 | 412.17186388950375 |Figure S1: Comparison of the overall yields for libraries prepared with the Truseq Nano kit with either the Sanger adaptors (original Illumina adaptors, in pink) and the modern Illumina adaptors (in blue).

## Slide 3
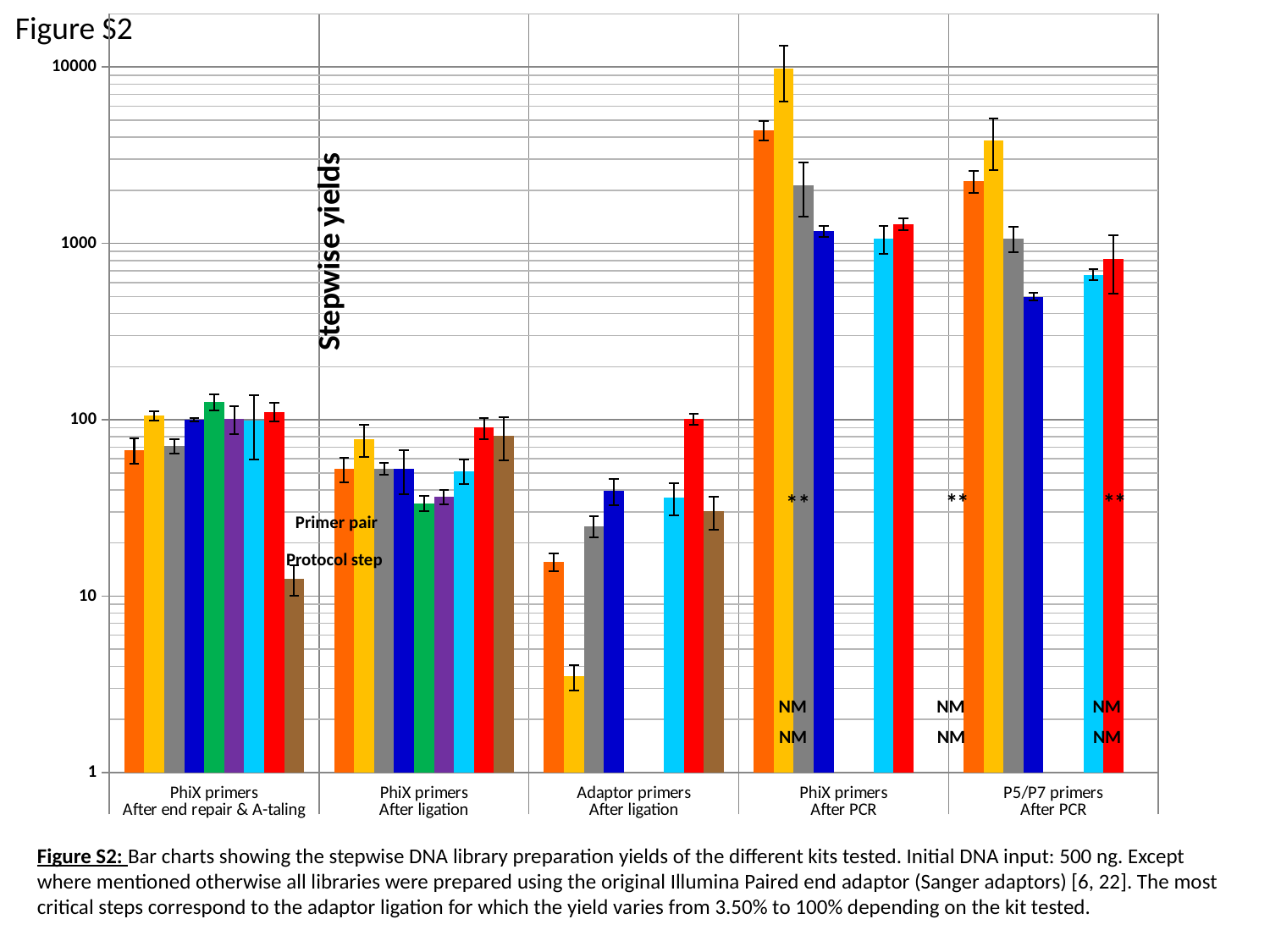

### Chart
| Category | NEBNext | NEBNext Ultra | SureSelect | Truseq Nano (Illumina adaptors) | Accel-NGS 1S (Swift adaptors) | Accel-NGS 2S (Swift adaptors) | Kapa Hyper | Kapa HyperPlus | Truseq DNA PCR-free (Illumina adaptors) |
|---|---|---|---|---|---|---|---|---|---|
| PhiX primers | 67.1423968490959 | 105.24510231042329 | 70.75945459951188 | 100.14915062081003 | 126.01484134330849 | 101.49713500822345 | 99.15064426336001 | 111.0 | 12.470735994798702 |
| PhiX primers | 52.425189420093716 | 77.84354774210946 | 52.977223852372894 | 52.63795335601004 | 33.627758538952385 | 36.53884564233163 | 51.14621815921334 | 90.10701933861209 | 80.9718885446651 |
| Adaptor primers | 15.66720734094684 | 3.498649508141112 | 24.98362390108301 | 39.55348214687396 | None | None | 36.13776250009588 | 100.8423403352704 | 30.266398716092407 |
| PhiX primers | 4392.838693013044 | 9805.789445163646 | 2144.852852410392 | 1171.641972820176 | None | None | 1060.5625752375324 | 1287.2876085454973 | None |
| P5/P7 primers | 2258.990525166426 | 3853.5728530268775 | 1067.4659966742802 | 498.5791495364397 | None | None | 665.460184092668 | 815.5482133750103 | None |*
*
*
*
*
*
Primer pair
Protocol step
NM
NM
NM
NM
NM
NM
Figure S2
Figure S2: Bar charts showing the stepwise DNA library preparation yields of the different kits tested. Initial DNA input: 500 ng. Except where mentioned otherwise all libraries were prepared using the original Illumina Paired end adaptor (Sanger adaptors) [6, 22]. The most critical steps correspond to the adaptor ligation for which the yield varies from 3.50% to 100% depending on the kit tested.

## Slide 4
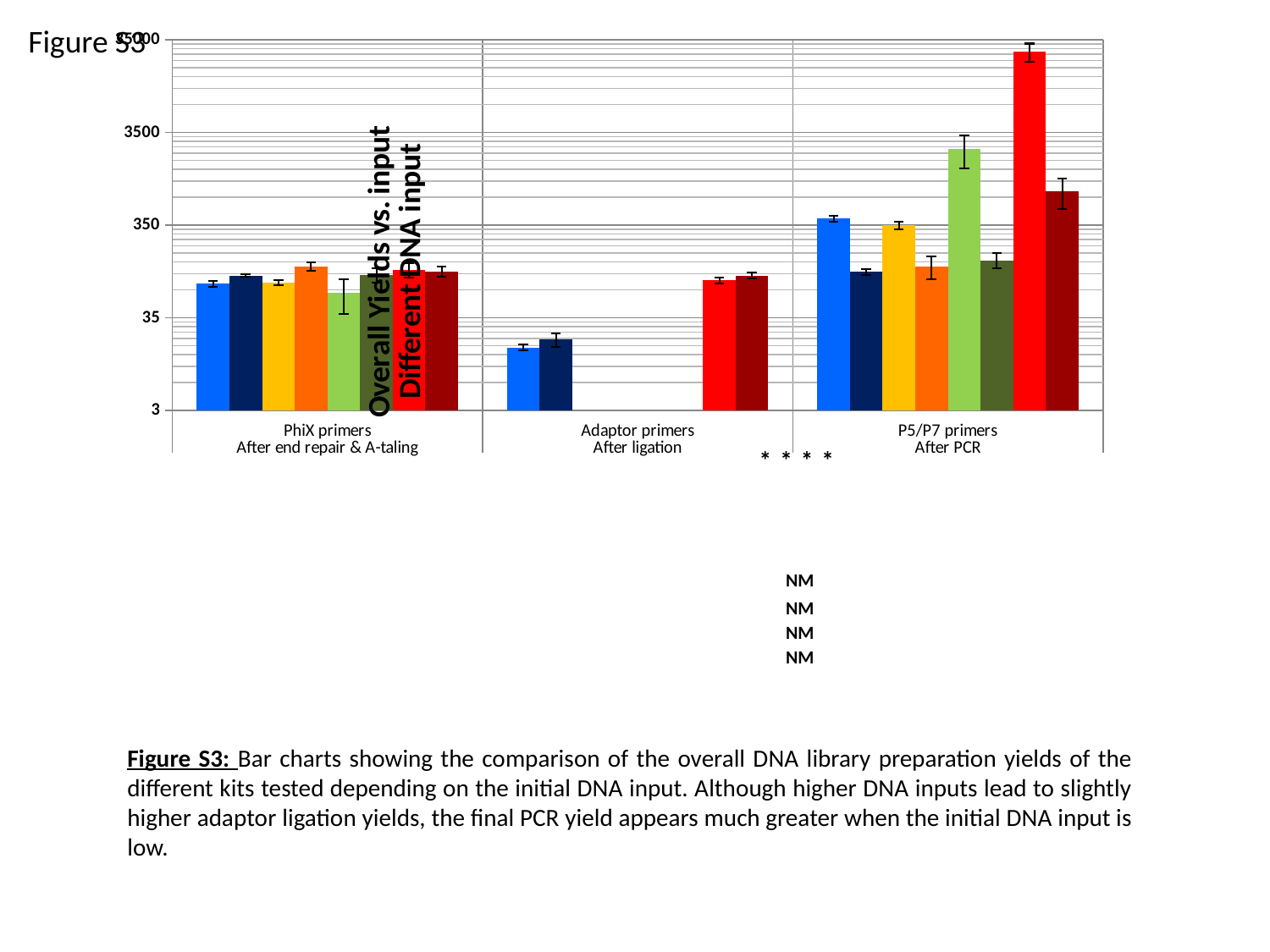

Figure S3
### Chart
| Category | Truseq Nano (100 ng input, Illumina adaptors) | Truseq Nano (500 ng input, Illumina adaptors) | 1S Swift Accel (100 ng input, Swift adaptors) | Accel-NGS 1S (500 ng input, Swift adaptors) | Accel-NGS 2S (20 ng input, Swift adaptors) | Accel-NGS 2S (500 ng input, Swift adaptors) | KAPA HyperPlus (20 ng input, Sanger adaptors) | KAPA HyperPlus (500 ng input, Sanger adaptors) |
|---|---|---|---|---|---|---|---|---|
| PhiX primers | 81.24160243484647 | 100.14915062081003 | 83.40224128877496 | 126.01484134330849 | 64.68409986188655 | 101.49713500822345 | 116.0 | 111.0 |
| Adaptor primers | 16.72105300488217 | 20.34066313660379 | None | None | None | None | 88.6948937845787 | 99.75510153625619 |
| P5/P7 primers | 412.17186388950375 | 109.16628281897744 | 349.8879090914627 | 124.84998895582838 | 2342.8141877578705 | 146.36348463754868 | 26000.620563761444 | 813.7695088083207 |*
*
*
*
NM
NM
NM
NM
Figure S3: Bar charts showing the comparison of the overall DNA library preparation yields of the different kits tested depending on the initial DNA input. Although higher DNA inputs lead to slightly higher adaptor ligation yields, the final PCR yield appears much greater when the initial DNA input is low.

## Slide 5
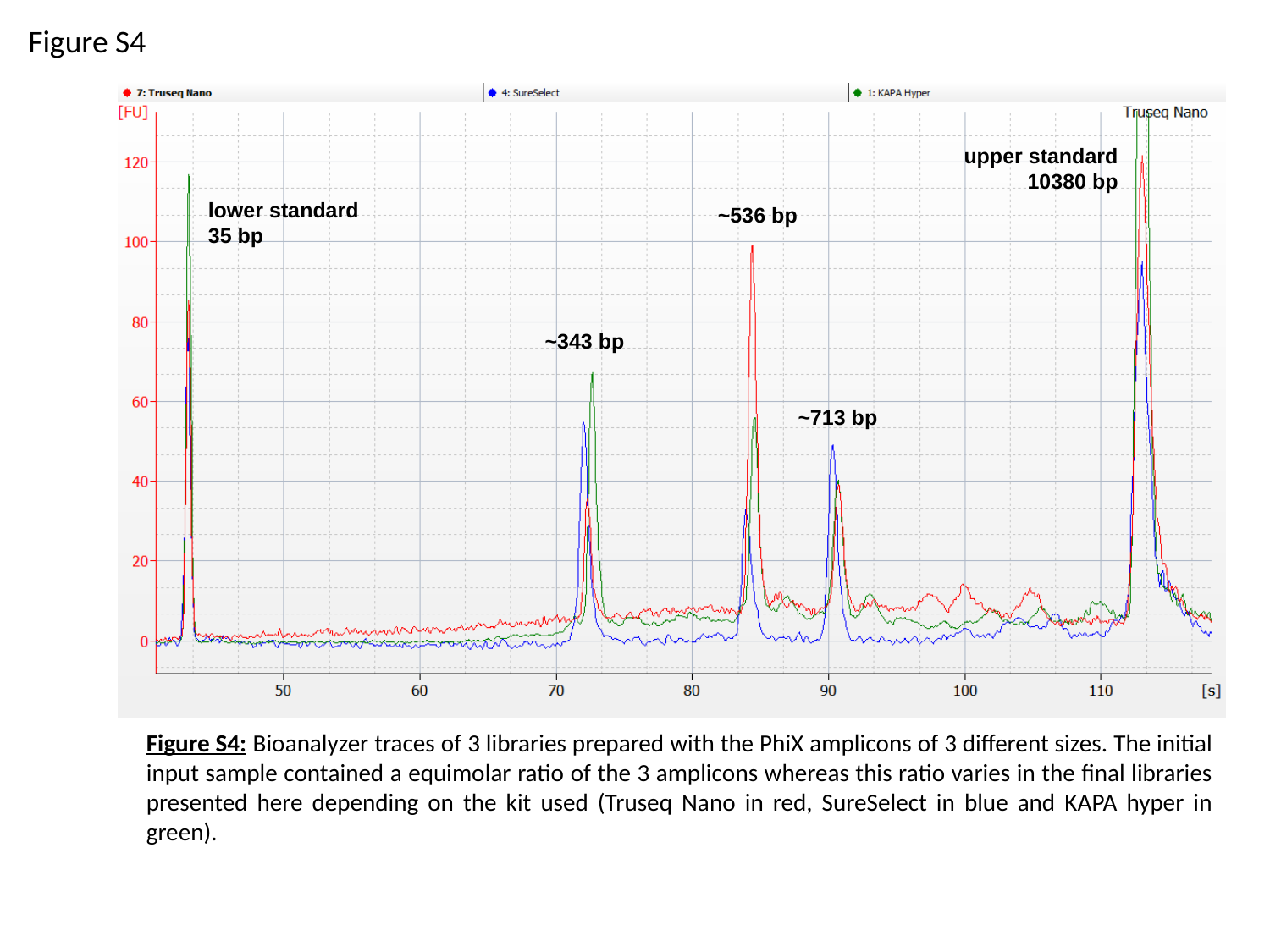

Figure S4
upper standard
10380 bp
lower standard
35 bp
~536 bp
~343 bp
~713 bp
Figure S4: Bioanalyzer traces of 3 libraries prepared with the PhiX amplicons of 3 different sizes. The initial input sample contained a equimolar ratio of the 3 amplicons whereas this ratio varies in the final libraries presented here depending on the kit used (Truseq Nano in red, SureSelect in blue and KAPA hyper in green).

## Slide 6
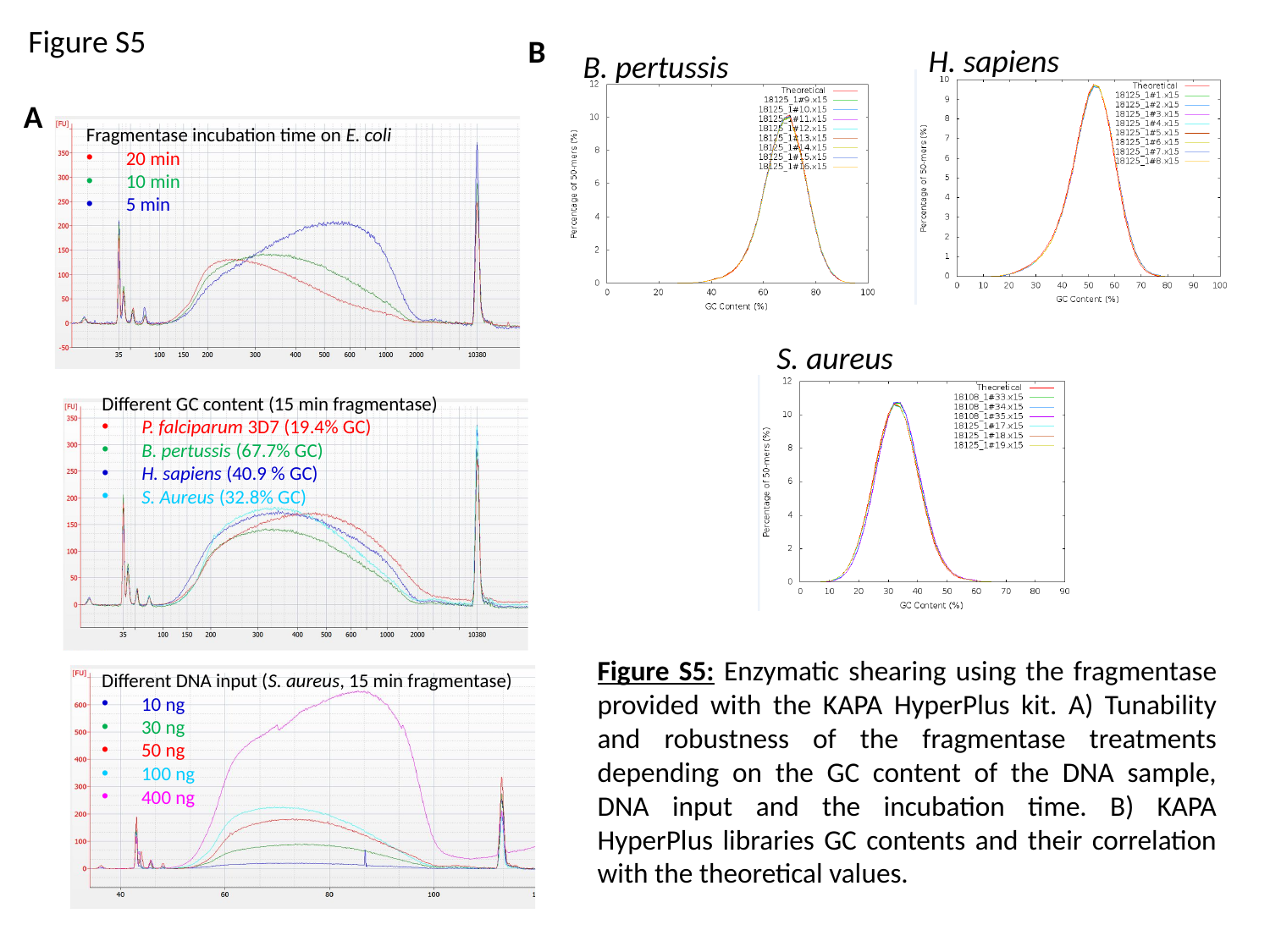

Figure S5
B
H. sapiens
B. pertussis
A
Fragmentase incubation time on E. coli
20 min
10 min
5 min
S. aureus
Different GC content (15 min fragmentase)
P. falciparum 3D7 (19.4% GC)
B. pertussis (67.7% GC)
H. sapiens (40.9 % GC)
S. Aureus (32.8% GC)
Figure S5: Enzymatic shearing using the fragmentase provided with the KAPA HyperPlus kit. A) Tunability and robustness of the fragmentase treatments depending on the GC content of the DNA sample, DNA input and the incubation time. B) KAPA HyperPlus libraries GC contents and their correlation with the theoretical values.
Different DNA input (S. aureus, 15 min fragmentase)
10 ng
30 ng
50 ng
100 ng
400 ng
