# Supplementary material for: Quantitation of next generation sequencing library preparation protocol efficiencies using droplet digital PCR assays - a systematic comparison of DNA library preparation kits for Illumina sequencing
Source: BMC Genomics. 2016 Jun 13;17:458. doi: 10.1186/s12864-016-2757-4 (PMC4906846; doi:10.1186/s12864-016-2757-4)
Supplement: Additional file 2: Table S1. — Example of dilution factors and yield calculations for the NEBNext libraries with 500 ng DNA input and Sanger adaptors. The calculation of the number of molecules measured by ddPCR at each step of the library preparation is described in the first 7 columns. The number 20 in column 7 corresponds to the ddPCR reaction volume in μL as only 1 μL of diluted sample is pipetted in the final ddPCR reaction mix of 20 μL total. Column 8 to 10 describe the calculation of the Overall Yield of each steps whereas column 11 to 14 (table split on 2 pages) explain the calculations of the Step Yield. The equations corresponding to each cell/column values are displayed in blue. (DOCX 22 kb) [file 12864_2016_2757_MOESM2_ESM.docx]

**Table S1: *Example of dilution factors and yield calculations for the NEBNext libraries with 500 ng DNA input and Sanger adaptors. The calculation of the number of molecules measured by ddPCR at each step of the library preparation is described in the first 7 columns. The number 20 in column 7 corresponds to the ddPCR reaction volume in µL as only 1 µL of diluted sample is pipetted in the final ddPCR reaction mix of 20 µL total. Column 8 to 10 describe the calculation of the Overall Yield of each steps whereas column 11 to 14 (table split on 2 pages) explain the calculations of the Step Yield. The equations corresponding to each cell/column values are displayed in blue.***

| **1** | **2** | **3** | **4** | **5** | **6** | **7** | **8** | **9** | **10** |
| --- | --- | --- | --- | --- | --- | --- | --- | --- | --- |
| **Triplicate number** | **Step** | **Primers** | **Sample dilution prior to ddPCR** | **Sample volume at that step of the library preparation (µL)** | **# of molecules in ddPCR well** | **Number of molecules at that step in the sample** | **Overall Yields (%)** | **Average Overall Yields (%)** | **Standard Dev. Overall Yield (%)** |
|  |  |  | *d* | *v* | *n* | *N = n * v * d * 20* | *Y = N_(step)_ * 100 / N_(covaris)_* | *Y_av._ = (Y_1_ + Y_2_ + Y_3_) / 3* | *Sdt(Av.Yield)* |
| 1 | Before Covaris | PhiX | 1E+06 | 130.0 | 370 | 9.62E+11 | 100.0 | 100 | 0 |
|  | After end repair & A-taling | PhiX | 1E+07 | 12.0 | 298 | 7.15E+11 | 74.4 | 67 | 11 |
|  | After ligation | PhiX | 1E+06 | 22.0 | 706 | 3.11E+11 | 32.3 | 35 | 4 |
|  | After ligation | Adaptor | 5E+05 | 22.0 | 232 | 5.10E+10 | 5.3 | 6 | 1 |
|  | After PCR | PhiX | 1E+07 | 30.0 | 379 | 2.27E+12 | 236.4 | 236 | 22 |
|  | After PCR | P5/P7 | 1E+06 | 30.0 | 1920 | 1.15E+12 | 119.8 | 121 | 9 |
| 2 | Before Covaris | PhiX | 1E+06 | 130.0 | 335 | 8.71E+11 | 100.0 |  |  |
|  | After end repair & A-taling | PhiX | 1E+07 | 12.0 | 264 | 6.34E+11 | 72.7 |  |  |
|  | After ligation | PhiX | 1E+06 | 22.0 | 788 | 3.47E+11 | 39.8 |  |  |
|  | After ligation | Adaptor | 5E+05 | 22.0 | 267 | 5.87E+10 | 6.7 |  |  |
|  | After PCR | PhiX | 1E+07 | 30.0 | 374 | 2.24E+12 | 257.6 |  |  |
|  | After PCR | P5/P7 | 1E+06 | 30.0 | 1890 | 1.13E+12 | 130.2 |  |  |
| 3 | Before Covaris | PhiX | 1E+06 | 130 | 344 | 8.94E+11 | 100.0 |  |  |
|  | After end repair & A-taling | PhiX | 1E+07 | 12 | 118 | 4.86E+11 | 54.3 |  |  |
|  | After ligation | PhiX | 1E+06 | 22 | 653 | 2.87E+11 | 32.1 |  |  |
|  | After ligation | Adaptor | 5E+05 | 22 | 178 | 3.92E+10 | 4.4 |  |  |
|  | After PCR | PhiX | 1E+07 | 30 | 320 | 1.92E+12 | 214.7 |  |  |
|  | After PCR | P5/P7 | 1E+06 | 30 | 1690 | 1.01E+12 | 113.4 |  |  |

| **1** | **2** | **3** | **7** | **11** | **12** | **13** | **14** |
| --- | --- | --- | --- | --- | --- | --- | --- |
| **Triplicate number** | **Step** | **Primers** | **Number of molecules at that step in the sample** | **Step Yields (%)** | **Step Yields Calculation** | **Average Step Yields (%)** | **Standard Dev. Step Yield (%)** |
|  |  |  | *N = n * v * d * 20* |  |  | *Y_av._ = (Y_1_ + Y_2_ + Y_3_) / 3* | *Sdt(Av.Yield)* |
| 1 | Before Covaris | PhiX | 9.62E+11 | 100.0 | *Y = N_(covaris, PhiX primers)_ * 100 / N_(covaris, PhiX primers)_* | 100 | 0 |
|  | After end repair & A-taling | PhiX | 7.15E+11 | 74.6 | *Y = N_(A-tail., PhiX primers)_ * 100 / N_(covaris, PhiX primers)_* | 67 | 11 |
|  | After ligation | PhiX | 3.11E+11 | 43.4 | *Y = N_(lig., PhiX primers)_ * 100 / N_(A-tail., PhiX primers)_* | 52 | 8 |
|  | After ligation | Adaptor | 5.10E+10 | 16.4 | *Y = N_(lig., Adaptor primers)_ * 100 / N_(lig., PhiX primers)_* | 16 | 2 |
|  | After PCR | PhiX | 2.27E+12 | 4455.3 | *Y = N_(PCR, PhiX primers)_ * 100 / N_(lig., PhiX primers)_* | 4393 | 544 |
|  | After PCR | P5/P7 | 1.15E+12 | 2257.1 | *Y = N_(PCR, P5/P7 primers)_ * 100 / N_(PCR, PhiX primers)_* | 2259 | 329 |
| 2 | Before Covaris | PhiX | 8.71E+11 | 100.0 |  |  |  |
|  | After end repair & A-taling | PhiX | 6.34E+11 | 72.7 |  |  |  |
|  | After ligation | PhiX | 3.47E+11 | 54.7 |  |  |  |
|  | After ligation | Adaptor | 5.87E+10 | 167.0 |  |  |  |
|  | After PCR | PhiX | 2.24E+12 | 3820.2 |  |  |  |
|  | After PCR | P5/P7 | 1.13E+12 | 1930.5 |  |  |  |
| 3 | Before Covaris | PhiX | 8.94E+11 | 100.0 |  |  |  |
|  | After end repair & A-taling | PhiX | 4.86E+11 | 54.3 |  |  |  |
|  | After ligation | PhiX | 2.87E+11 | 59.1 |  |  |  |
|  | After ligation | Adaptor | 3.92E+10 | 13.6 |  |  |  |
|  | After PCR | PhiX | 1.92E+12 | 4903.0 |  |  |  |
|  | After PCR | P5/P7 | 1.01E+12 | 2589.4 |  |  |  |
